# 545. A Retrospective Review of 30-day Mortalities in Solid-Organ Transplant Recipients (SOT) versus Non-Transplant Patients (NTP) Receiving Remdesivir (REM) and Dexamethasone (DEX) for COVID-19 Pneumonia

**DOI:** 10.1093/ofid/ofab466.744

**Published:** 2021-12-04

**Authors:** Krina Vyas, Kevin L Epps, Nan Zhang, Sadia Shah, Matthew Soto-Arenall, Lisa Brumble, Claudia R Libertin

**Affiliations:** Mayo Clinic, Jacksonville, Jacksonville, FL

## Abstract

**Background:**

Treating COVID-19 infection in SOT is challenging due to long-term use of immunosuppressive agents. REM is the only FDA-approved anti-viral for SARS-COV-2 infection. DEX showed decrease in mortality in the Recovery Trial. COVID-19 treatment guidelines for SOT patients are the same as NTP despite limited literature on those outcomes. Our primary objective was to determine if 30-day mortality was different between SOT and NTP matched cohorts using these 2 drugs. The secondary objectives included comparisons of length of stay (LOS), days on mechanical ventilation (DMV), and the use of other treatment modalities.

**Methods:**

We retrospectively collected data for hospitalized SOT and NTP, 18 years and older, with pcr-confirmed SARS-CoV-2 infection receiving REM and DEX from May 1, 2020, to October 10, 2020, at Mayo Clinic Florida. IRB approval was obtained. Descriptive statistics were used to analyze the data. Continuous variables were summarized as mean (standard deviation) or median (range) where appropriate, while categorical variables were reported as frequency (percentage).

**Results:**

Of 80 patients who met the inclusion criteria, 28 were SOT, and 52 were NTP. The SOT cohort was subcategorized below:

SOT patients were significantly younger than NTP (p < .001). Further, SOT patients had significantly longer LOS (p = 0.043) and more COVID-19 modalities (75% vs. 36.5%, p = 0.002) compared to NTP. Among the 28 SOT patients, 2 of them died within 30 days of admission, and among the 52 NTP patients, 7 of them died within 30 days. The 30-d survival estimate for SOT group is 92.9% (95% CI: 83.8% - 100.0%) and for NTP group is 86.5% (95% CI: 77.7% - 96.3%). The log-rank test was not significant between the groups (p=0.37), but the NTP has a worse survival curve from the figure below.

SOT-NTP Survival Curve

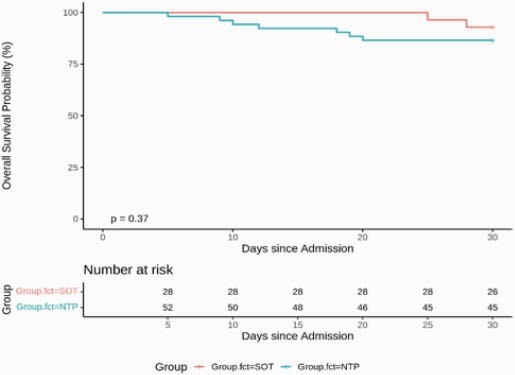

**Conclusion:**

SOT group was younger, had longer LOS, and more COVID-related modalities. The 30-d survival estimate for SOT group is 92.9% and for NTP group is 86.5%, but the survival curve for NTP was worse likely secondary to age. Use of REM & DEX in SOT recipients is a valid recommendation.

**Disclosures:**

**Claudia R. Libertin, MD**, **Gilead** (Grant/Research Support)

**Table:** 

Characteristics	Number of SOT (n = 28)
Transplant Type	
Kidney n (%)	15 (53.7)
Lung n (%)	3 (10.7)
Liver n (%)	2 (7.1)
Heart n (%)	6 (21.4)
Multi-organ n (%)	2 (7.1)

